# Identification of individual conformers in C_4_H_6_O isomers using conformer-specific vibrational spectroscopy†

**DOI:** 10.1039/d1ra07397d

**Published:** 2021-11-29

**Authors:** Sung Man Park, Chan Ho Kwon

**Affiliations:** Department of Chemistry, Institute for Molecular Science and Fusion Technology, Kangwon National University Chuncheon 24341 Korea chkwon@kangwon.ac.kr

## Abstract

We measured the conformer-specific vibrational spectra of C_4_H_6_O isomers in neutral and cationic states using IR resonant vacuum ultraviolet mass-analyzed threshold ionization (VUV-MATI) spectroscopy for the first time. Notably, the measured IR dip and hole-burn VUV-MATI spectra for each isomer represent the identifiable vibrational spectra of individual conformers in both states. Furthermore, we estimated the relative populations of individual conformers in crotonaldehyde (CA) and methyl vinyl ketone (MVK) isomers using the IR dip intensity, the corresponding Franck–Condon factor, and the IR absorption cross section. Our analysis revealed that the compositional ratio of s-*trans* to s-*cis* conformers in the CA isomer remained at 95.8 : 4.2 even under supersonic expansion, whereas that in the MVK isomer was determined as 90.6 : 9.4, which is consistent with previous research. These findings reveal that the conformational stability of each isomer depends on the position of the methyl group relative to the carbonyl group.

## Abstract 2

We measured the conformer-specific vibrational spectra of C_4_H_6_O isomers in neutral and cationic states using IR resonant vacuum ultraviolet mass-analyzed threshold ionization (VUV-MATI) spectroscopy for the first time.

## Graphical abstract

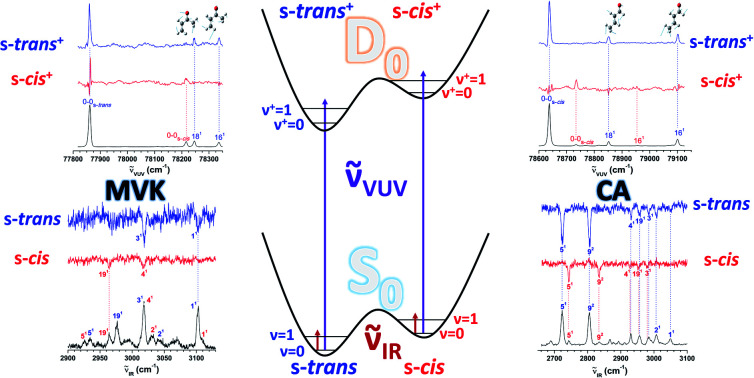

## Introduction

The identification of individual conformers is typically performed using unique spectroscopic techniques capable of providing insights into the conformation-dependent reactivity of specific chemical reactions.^1–4^ Such techniques reduce conformational complexity by utilizing the adiabatic cooling of molecules that results from supersonic expansion in the absence of the non-equilibrium kinetic effect.^5,6^ Subsequent investigations have revealed that molecules with a sufficiently low conformer interconversion barrier exist in conformational equilibrium even at low temperatures,^7–9^ whereas conformers with a high interconversion barrier retain their original composition during cooling *via* supersonic expansion.^10–12^ For the former case, identifying peaks that correspond to a specific conformer in measured vibrational spectra requires additional exploration of the vibrational temperature-dependent conformational population, which, in turn, depends on the constructed potential energy surfaces associated with conformational interconversion. Nevertheless, deciphering the contribution of each conformer in the congested vibrational spectrum of a polyatomic molecule is still immensely difficult because of the comparable force fields between the atoms in conformers. As such, laser double resonance techniques such as hole-burning and IR depletion spectroscopies^13,14^ have been suggested as alternative approaches to characterize the structures of individual conformers, although these techniques focus mainly on hydrogen-bonded clusters or van der Waals complexes in chromophore-containing systems, where resonance-enhanced multiphoton ionization (REMPI) is typically adopted.^15–18^ Meanwhile, for analyzing aliphatic species without an ultraviolet (UV) chromophore group, the IR vacuum ultraviolet (VUV) double resonance techniques that circumvent the limitations of REMPI have been developed, although excess VUV photon energy at 118 nm (the wavelength normally used) may cause uncontrollable fragmentations.^19–21^

α,β-Unsaturated carbonyl compounds that consist of an ethene conjugated to a carbonyl group, providing them with relatively high electrophilicity, have been researched extensively as an important precursor in reactions with nucleophiles as well as in astrochemical-relevant models of the interstellar medium.^22–25^ Interestingly, even simple precursors such as acrolein exhibit interconversion between s-*trans* and s-*cis* conformers, which manifests as rotation along the single bonds between ethene and carbonyl groups in the molecule. In principle, although the stereoselectivities of reactions depend on multiple environmental factors, the reaction pathway is affected primarily by the conformational preference in molecules, as described by the Diels–Alder reaction.^23^ In addition, it has been reported that compounds with acrolein moiety undergo a variety of photochemical processes when in an excited state, which have prompted attempts to elucidate the photochemistry of conjugated enone molecules.^26–28^ These subsequent studies indicate that the photoproducts can be governed by the conformational preference of the designated molecule in an electronic state. Therefore, the conformation responsible for photoreaction should be understood in terms of factors such as molecular orbital interactions and stabilization energies, which can be calculated by various theoretical models.^29,30^

**Fig. 1:**
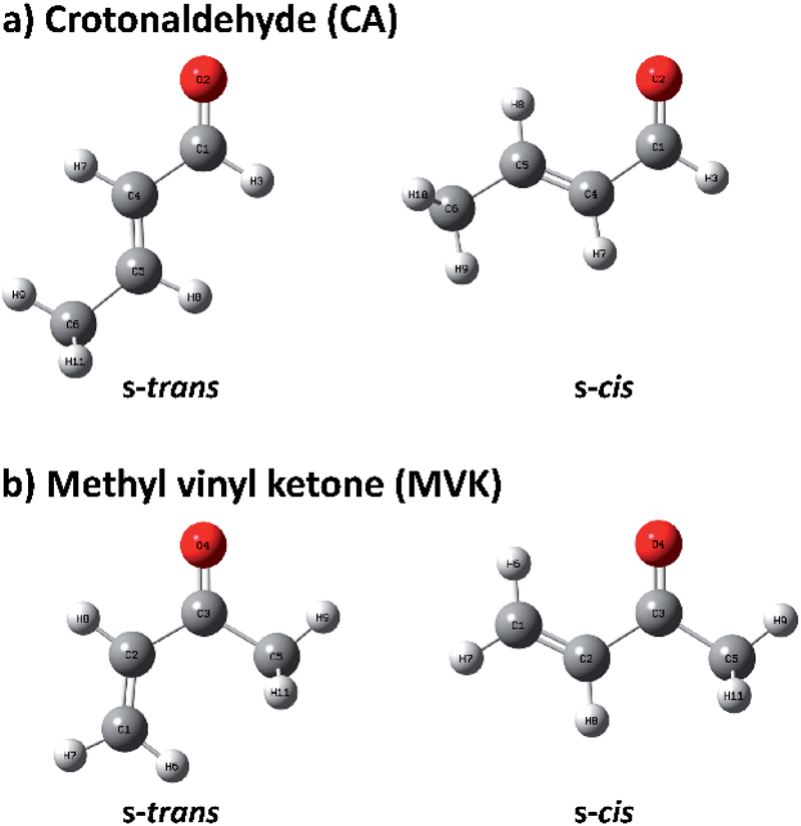
Geometries of s-*trans* and s-*cis* conformers in (a) CA and (b) MVK isomers.

Because of the importance of the methyl-substituted acrolein in both synthetic and atmospheric chemistry, the conformational stabilities and structures of s-*trans* and s-*cis* conformers are usually studied by substituting a methyl group in acrolein with crotonaldehyde (CA) and methyl vinyl ketone (MVK), of which conformational geometries are shown in Fig. 1.^31–34^ Before recent studies using single-photon vacuum ultraviolet mass-analyzed threshold ionization (VUV-MATI) spectroscopy and the Franck–Condon (FC) simulations,^9,10^ most investigations involving the conformations of these two isomers only examined the temperature-dependence of the peaks corresponding to each neutral conformer in vibrational or rotational spectra. This contributed to the fact that CA and MVK have very short lifetimes, that is, a few hundred femtoseconds, in the excited electronic state owing to the substituent effect of the methyl group on electronic relaxation rates.^35^ Most spectral analyses are consistent in finding that the s-*trans* conformer is more stable than the s-*cis* conformer despite the deviations in their relative stabilities. However, the theoretical predictions regarding the preferential conformation for MVK were inconsistent with the recent experimental results, suggesting that the uncertainty in the calculated energy values can originate from the use of incorrect functions and/or densities.^9^ Consequently, we were motivated to obtain identifiable vibrational spectra of individual conformers (*i.e.*, s-*trans* and s-*cis*) in the two isomers, and thus provide indisputable experimental data regarding the conformational populations while also elucidating how the conformational stabilities in the two isomers change relative to the position of the methyl group.

## Experimental

Experiments were performed using a homebuilt VUV-MATI spectrometer, the details of which have been provided in previous studies.^36,37^ A coherent and tunable VUV laser pulse with a wavelength range of 126.3–128.6 nm was generated by resonant four-wave difference frequency mixing (FWDFM) based on 4p^6^–5p[1/2]_0_ or 4p^6^–5p[5/2]_2_ transitions in Kr, which were alternated to avoid wavevector mismatches depending on the VUV wavelength. The MATI ion signals were normalized using the power of the tunable visible laser that was used for the FWDM-based VUV generation. For the IR resonant VUV-PI/MATI scheme, high-resolution IR laser light in the range of 2670–3100 cm^−1^ with an approximate energy of 7 mJ per pulse was generated using a narrowband optical parametric oscillator/amplifier (OPO/OPA) system (LaserVision, ∼0.1 cm^−1^) pumped by a Nd:YAG laser seeded with a wavelength of 1064 nm. Next, the generated IR laser light was cylindrically focused using a telescope consisting of two CaF_2_ lenses (*f* = 75 and −100 mm) and aligned perpendicularly to both the molecular beam and the ion-flight direction into the photoionization chamber. The VUV laser pulse was delayed by approximately 5 ns with respect to the IR laser pulse, which photoexcites the neutral conformers to a vibrational state. The frequencies of all lasers used in the experiments were measured and calibrated using a wavemeter (HighFinesse, Wavelength Meter WS5) with an accuracy of 0.1 cm^−1^.

## Results and discussion

**Fig. 2:**
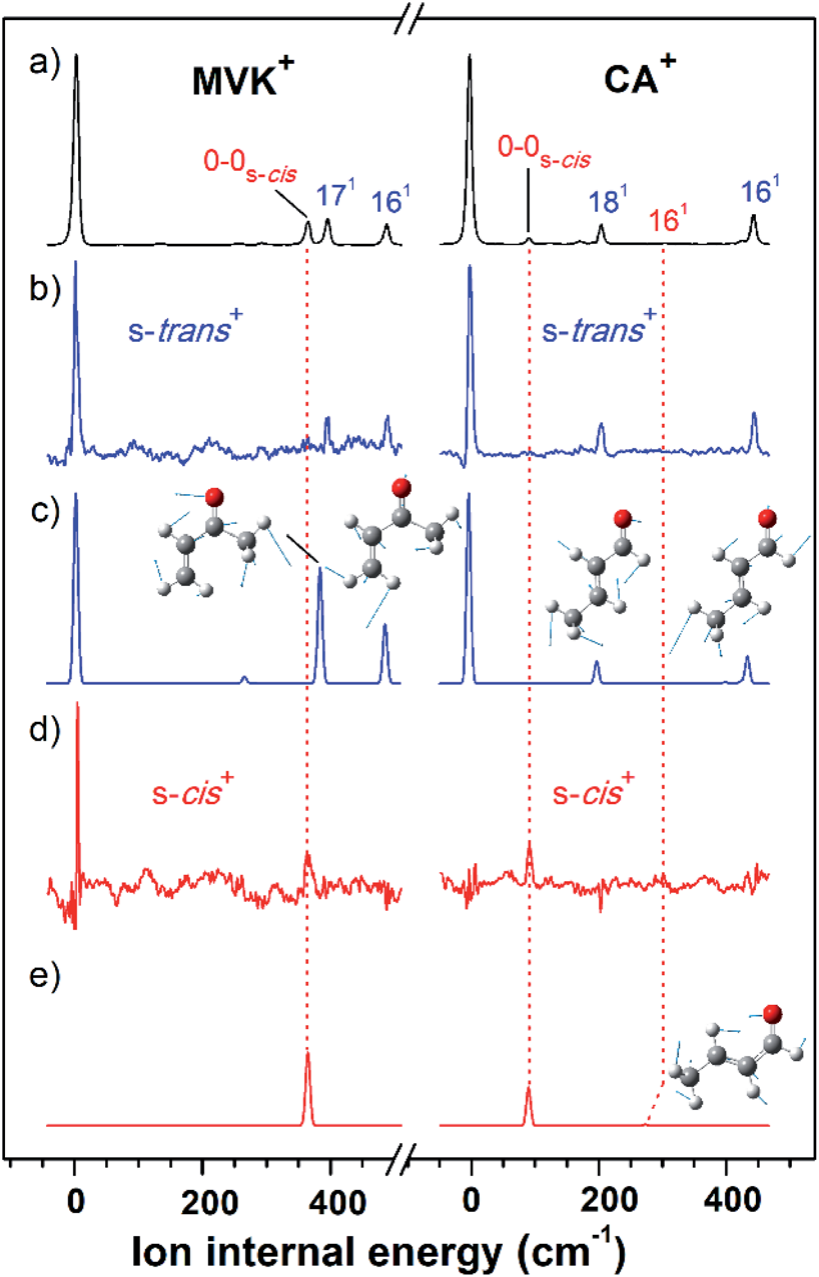
(a) One-photon VUV-MATI spectra of MVK (left side) and CA (right side). The ion internal energy along the bottom horizontal axis corresponds to the vibrational frequency of each s-*trans* conformer in MVK and CA, which was estimated using the energy relative to each original band. (b) IR hole-burn VUV-MATI spectra of MVK (left side) and CA (right side) measured by exciting the vibrational modes of the s-*trans* conformers in MVK and CA, which occur at 3103 and 2724 cm^−1^, respectively. (c) FC-simulated spectra of the s-*trans* conformers in MVK (left side) and CA (right side). (d) IR hole-burn VUV-MATI spectra of MVK (left side) and CA (right side) measured by exciting the vibrational modes of the s-*cis* conformers in MVK and CA, which occur at 2964 and 2745 cm^−1^, respectively. (e) FC-simulated spectra of s-*cis* conformers in MVK (left side) and CA (right side). All spectra were normalized with respect to the 0–0 band of each conformer, except for in (d) and (e), where a four-fold magnification was applied to the 0–0 bands of the s-*cis* conformers for comparison.

**Fig. 3:**
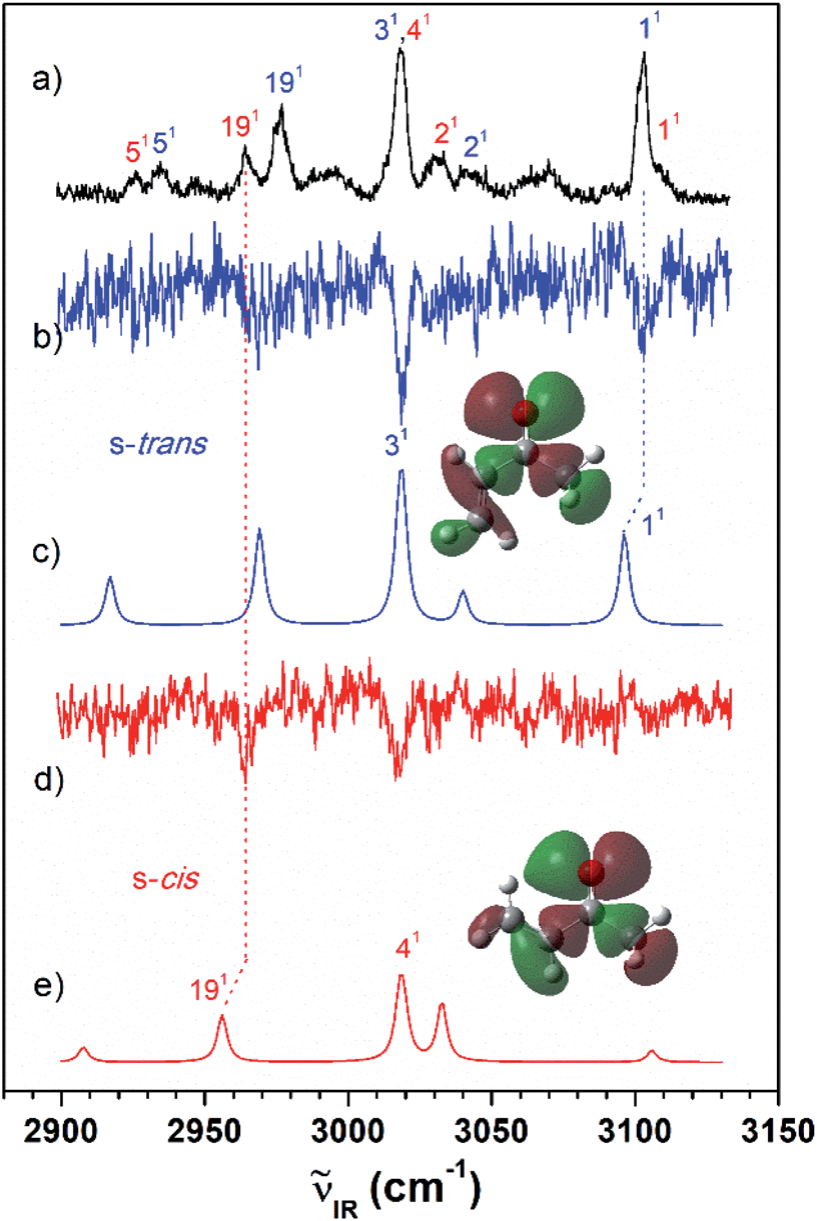
(a) IR resonant VUV (77 762 cm^−1^) photoionization spectra of MVK measured by scanning the IR laser. The IR dip VUV-MATI spectra were measured by monitoring the origin bands expected for (b) s-*trans* and (d) s-*cis* conformers (77 861 and 78 216 cm^−1^, respectively). Simulated IR spectra of the (c) s-*trans* and (e) s-*cis* conformers calculated at the B3LYP/aug-cc-pVTZ level, which provide the best agreement among the various calculation results, as shown in Fig. S1.† Spectra for the s-*trans* and s-*cis* conformers were normalized with respect to the 3-mode and 19-mode intensities, respectively. The highest occupied molecular orbitals of the (c) s-*trans* and (e) s-*cis* conformers were inserted in the simulated IR spectra to compare the hyperconjugation-related stabilization between the σ orbitals and the lone-pair p orbital of the oxygen atoms.

**Fig. 4:**
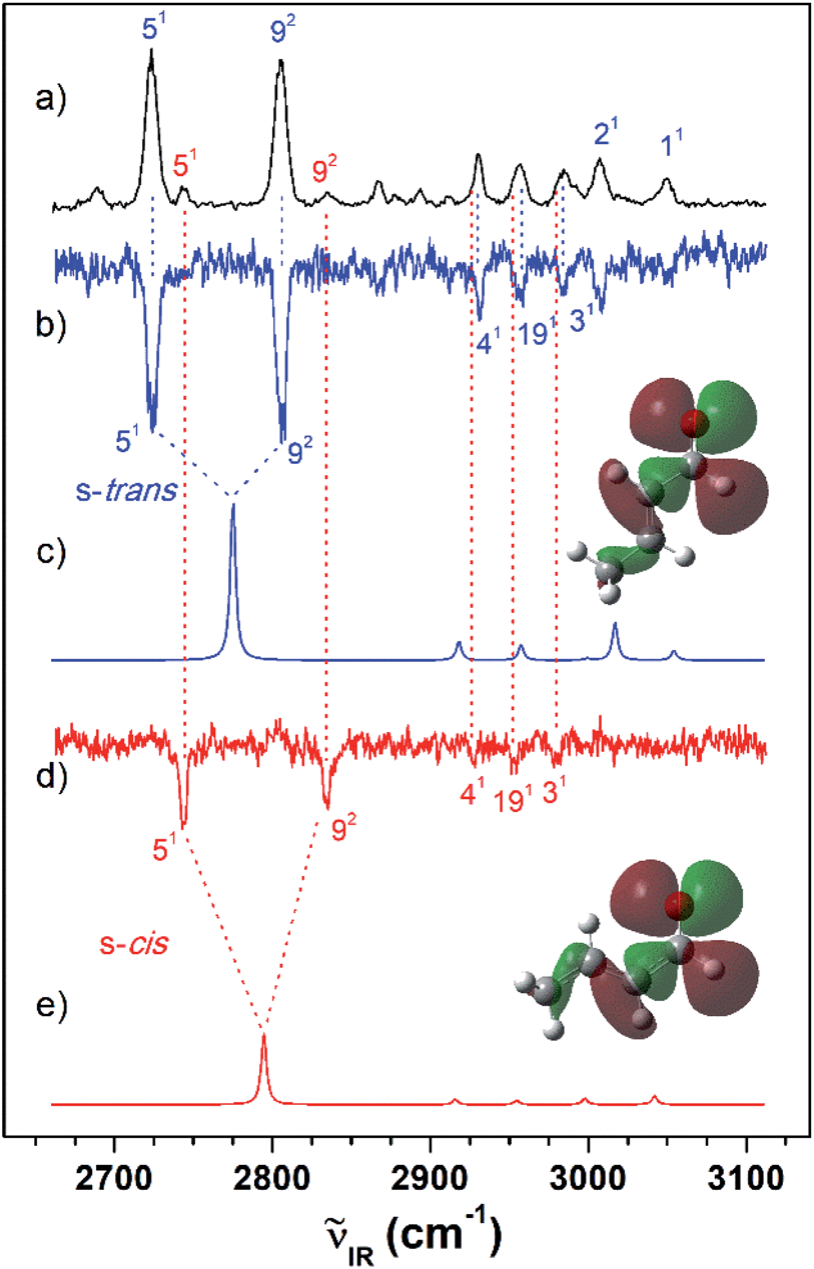
(a) IR resonant VUV (78 540 cm^−1^) photoionization spectra of CA measured by scanning the IR laser. IR dip VUV-MATI spectra measured by monitoring the origin bands expected for (b) s-*trans* and (d) s-*cis* conformers (78 638 and 78 734 cm^−1^, respectively). Simulated IR spectra of the (c) s-*trans* and (e) s-*cis* conformers calculated at the B3LYP/aug-cc-pVTZ level, which provide the best agreement among the various calculation results, as shown in Fig. S2.† Spectra for the s-*trans* conformer were normalized with respect to the 5-mode intensity, whereas spectra for the s-*cis* conformer were scaled by a factor of four with respect to the 5-mode intensity for ease of comparison. The highest occupied molecular orbitals of the (c) s-*trans* and (e) s-*cis* conformers were inserted in the simulated IR spectra to compare the hyperconjugation-related stabilization between the σ orbitals and the lone-pair p orbital of the oxygen atoms.

As shown in Fig. 2(a), the VUV-MATI spectra of the MVK and CA isomers, which are essentially the vibrational spectra of the two isomers in the D_0_ state, were measured as a function of the VUV photon energy. The two most intense peaks, at 77 861 and 78 638 cm^−1^ correspond to the 0–0 bands of MVK and CA, respectively, which is consistent with the respective adiabatic ionization energy (AIE) values of 77 867 ± 4 and 78 640 ± 3 cm^−1^ determined in previous studies by extrapolating to the zero-field limit of the MVK and CA using VUV-MATI spectroscopy.^9,10^ At present, other than using FC simulations to determine the ionic transitions of the conformers expected in the S_0_ state, no method exists for identifying the vibrational peaks corresponding to individual conformers in the MATI spectrum of each isomer. In addition, the IR absorption spectra of the MVK and CA were obtained by utilizing the IR resonant VUV-PI scheme, where the frequency of the IR laser pulse preceding the VUV laser pulse fixed at 77 762 or 78 540 cm^−1^, *i.e.*, below the ionization thresholds of MVK and CA, respectively, was scanned. The IR absorption spectra of the MVK and CA in the S_0_ state are shown in Fig. 3(a) and 4(a), respectively. The vibrational peaks corresponding to individual conformers in the IR absorption spectrum of each isomer might be identified using the simulated IR spectrum of each conformer in the S_0_ state, with this process repeated for the MATI spectra. Accordingly, the conformer-specific vibrational spectra of the neutral isomer, that is, the IR dip VUV-MATI spectra of MVK and CA, were recorded by monitoring the 0–0 bands at 77 861 and 78 638 cm^−1^ that were observed in the VUV-MATI spectra while scanning the frequency of the IR laser (Fig. 3(b) and 4(b)). The measured IR dip spectra are expected to be a proxy for the vibrational spectra of the stable s-*trans* conformers for the two isomers in the S_0_ state. To check this, the IR spectra of the s-*trans* and the s-*cis* conformers in each isomer were simulated at various calculation levels using the aug-cc-pVTZ basis set,^38^ as shown in Fig. S1 and S2.† All simulated vibrational frequencies were scaled to compensate for the uncertainties caused by the vibrational anharmonicity and the incomplete treatment of electron correlation, which are artifacts of using finite basis sets.^39,40^ Based on the simulated IR spectra (Fig. 3(c) and 4(c)) that agreed most closely with the experimental spectra, peaks observed in the IR dip spectra can be successfully assigned to the fundamental vibrational modes characterizing the C–H stretching vibrations of the s-*trans* conformer of each isomer (Table 1). As mentioned above, this implies that the measured IR dip spectra correspond to the identifiable vibrational spectra of the s-*trans* conformers for the two isomers in the S_0_ state.

**Table 1:** Vibrational assignment (in cm^−1^) of individual conformers for the MVK and CA isomers in the S_0_ state

Mode^a^	IR + VUV	IR dip	Ref^b^	Calc^c^	Mode description^d^
MVK					

s-*trans* (*C*_s_)					
5^1^	2934		2949	2916	CH_3_*sym* stretch
19^1^	2977		2980	2969	Out-of-plane methyl CH stretch
3^1^	3018	3018	3018	3019	In-phase terminal CH_2_ stretch
2^1^	3042		3072	3040	Central C–H stretch
1^1^	3103	3103	3104	3096	Out-of-phase terminal CH_2_ stretch

s-*cis* (*C*_s_)					
5^1^	2925		2935	2908	CH_3_*sym* stretch
19^1^	2964	2964	2969	2956	Out-of-phase methyl CH stretch
4^1^	3018	3017	3020	3018	Out-of-phase vinyl CH stretch
2^1^	3030		3061	3033	Vinyl C–H breathing
1^1^	3109		3109	3106	Out-of-phase terminal CH_2_ stretch
CA					

s-*trans* (*C*_s_)					
5^1^	2724	2724	2722	2775^e^	Carbonyl CH stretch
9^2^	2805	2805	2805	2741^e^	Overtone of carbonyl CH bending
4^1^	2931	2931	2932	2918	CH_3_*sym* stretch
19^1^	2957	2957	2958	2957	Out-of-phase methyl CH stretch
3^1^	2986	2986	2990	2999	In-phase methyl and α-CH stretch
2^1^	3007	3007	3008	3017	Out-of-phase methyl and α-CH stretch
1^1^	3048		3050	3054	α-C–C(O)–H stretch

s-*cis* (*C*_s_)					
5^1^		2745		2795^e^	Carbonyl CH stretch
9^2^		2834		2768^e^	Overtone of carbonyl CH bending
4^1^		2928		2916	CH3 *sym* stretch
19^1^		2953		2955	Out-of-phase methyl CH stretch
3^1^		2980		2998	In-phase methyl and α-CH stretch

a Mulliken notation.

b Gas-phase IR spectrum from ref. 33.

c Harmonic vibrational frequencies calculated from the optimized geometries with *C*_s_ symmetry at the B3LYP/aug-cc-pVTZ level.

d Vibrational assignment reported in ref. 33.

e Peaks split by the Fermi resonance between the carbonyl CH stretching and overtone of carbonyl CH bending.

Furthermore, the distinct peaks observed at 3018 and 3103 cm^−1^ in the IR dip spectrum of the s-*trans* conformer in MVK (Fig. 3(b)) matched closely with the peaks observed in the IR absorption spectrum of MVK (Fig. 2(a)). The prominent peaks at 2724, 2805, 2931, 2957 and 3007 cm^−1^ in the IR dip spectrum of the s-*trans* conformer in CA (Fig. 4(b)) are also observed in Fig. 4(a). Notably, previous research indicates that the splitting of the two intense peaks at 2724 and 2805 cm^−1^ corresponds to the Fermi resonance between the C(O)–H stretching and the overtone of C(O)–H bending.^33,41,42^

The characteristic peaks (*i.e.*, the 1(a′) and 5(a′) modes) at 3103 and 2724 cm^−1^ isolated in the IR dip spectra of the s-*trans* conformers in MVK and CA were utilized in the IR hole-burn VUV-MATI scheme to acquire the identifiable vibrational spectra of the s-*trans* conformers for the two isomers in the D_0_ state. This scheme is based on the fact that when the frequency of the IR laser was fixed at the characteristic peak of the s-*trans* conformer in the S_0_ state, the corresponding MATI ion signals decrease owing to the vibrational excitation-induced reduction in the initial population of the s-*trans* conformer. In reality, the vibrational excitations for the s-*trans* conformers in the two isomers caused the 0–0 band signals for MVK and CA to be depleted by 5.3% and 8.0%, respectively, which confirms that the two 0–0 bands in the MATI spectra correspond to those of the s-*trans* conformers in the two isomers. For each isomer, the IR hole-burn spectrum of the s-*trans* conformer was obtained from the difference in the VUV-MATI spectra of the isomer with and without IR hole-burn laser irradiation, as shown in Fig. 2(b). The FC simulations for the s-*trans* conformer of each isomer in the D_0_ state (Fig. 2(c)) confirm that the measured IR hole-burn spectrum of each isomer represents the identifiable vibrational spectrum of each s-*trans* conformer in the D_0_ state. Thus, by comparing the IR hole-burn spectra of the s-*trans* conformers with the VUV-MATI spectra of the two isomers, it was found that the distinct peaks at 78 217 and 78 734 cm^−1^ in the MATI spectra were absent from the IR hole-burn spectra of the s-*trans* conformers, indicating that those peaks correspond to the s-*cis* conformers for the two isomers in the D_0_ state.

To monitor these peaks in the VUV-MATI spectra of MVK and CA, we measured the IR dip spectra while scanning the frequency of the IR laser, as shown in Fig. 3(d) and 4(d), respectively. As expected, the measured IR dip spectra corresponded to the IR spectra of the s-*cis* conformers in the S_0_ state for the two isomers, which were supported by the simulated IR spectra of the s-*cis* conformers in the two isomers shown in Fig. 3(e) and 4(e). The peaks in the measured IR dip spectra were attributed to the CH stretching vibrations of the s-*cis* conformers of each isomer (Table 1). As for the s-*trans* conformer, the Fermi resonance-related splitting at two similar frequency vibrations was observed at 2745 and 2834 cm^−1^. Remarkably, the IR absorption spectra of the MVK and CA (Fig. 3(a) and 4(a)) represent the summed IR dip spectra of the individual conformers, that is, the s-*trans* and the s-*cis* conformers for each isomer in the S_0_ state, which implies that the measured IR dip spectra of the two conformers in each isomer can be determined accurately from the IR absorption spectrum of each isomer.

As for the s-*trans* conformer, the IR hole-burn spectra of the s-*cis* conformers in the MVK and CA were acquired by utilizing the characteristic peaks (*i.e.*, the 19(a′) and 5(a′) modes) at 2964 and 2745 cm^−1^ isolated in the IR dip spectra of the s-*cis* conformers for the MVK and CA isomers in the S_0_ state. Despite their low intensity, distinct peaks corresponding to the 0–0 bands of the s-*cis* conformers were observed in the IR hole-burn spectra of the MVK and CA at 78 217 and 78 734 cm^−1^, respectively (Fig. 2(d)). In addition, the 0–0 band positions for the s-*cis* conformers in the two isomers corroborated the AIE values determined in previous studies.^9,10^ The assignments of the peaks observed in the IR hole-burn spectra of the individual conformers in the two isomers are listed in Table 2 alongside the calculated results and previously reported values. Owing to the vibrational excitations for the s-*cis* conformers of MVK and CA in the S_0_ state, the 0–0 band signals were depleted by 4.3% and 20.9%, respectively.

**Table 2:** Vibrational assignment (in cm^−1^) of individual conformers for MVK and CA in the D_0_ state

Mode^a^	IR hole-burn^b^	Ref^c^	Calc^d^	Mode description^e^
MVK				

s-*trans* (*C*_s_)				
0–0	77 861 (0)	77 867 ± 4 (0)		0–0 band
17^1^	78 246 (385)	78 253 (386)	375	In plane C–C=O bend
16^1^	78 336 (475)	78 342 (475)	475	In plane C=C–C bend
s-*cis* (*C*_s_)				
0–0	78 216 (355)	78 222 ± 4 (355)		0–0 band
CA				

s-*trans* (*C*_s_)				
0–0	78 638 (0)	78 640 ± 3 (0)		0–0 band
18^1^	78 851 (213)	78 855 (215)	208	Out-of-phase C–C=C–C bend
16^1^	79 101 (463)	79 103 (463)	453	In-phase C–C=C–C bend

s-*cis* (*C*_s_)				
0–0	78 734 (96)	78 736 ± 3 (96)		0–0 band
18^1^	78 953 (315)	78 984 (344)	286	In plane C=C–C bend

a Mulliken notation.

b Values in parentheses indicate the ion internal energies of vibrational peaks estimated with respect to the 0–0 band positions of individual conformers.

c Ref. 9 and 10 for MVK and CA, respectively.

d Harmonic vibrational frequencies calculated from the optimized geometries with *C*_s_ symmetry at B3LYP/aug-cc-pVTZ level.

e Vibrational assignments for MVK and CA reported in ref. 9 and 10, respectively.

We used the conformer-specific vibrational spectra of the MVK and CA isomers measured *via* IR dip and hole-burn VUV-MATI spectroscopy to estimate the relative amounts of the s-*trans* and the s-*cis* conformers in the S_0_ state for MVK and CA. When a molecule absorbs the IR photon of high frequency, the intramolecular vibrational relaxation (IVR) from the initially excited vibrational mode can occur to other low-frequency modes. Hence, we utilized the sharp dips in the IR dip VUV-MATI spectra so that the possibility of the IVR process is maximally excluded. Then, assuming that the vibrational excitation-induced conformer population reduction determined *via* IR absorption reflects the hole-burn in the MATI ion signal produced by the conformer, the relative populations of two conformers can be determined using the dip intensities in their respective IR dip spectra; these intensities should be divided by the FC-factor related to the 0–0 band and the IR absorption probability of vibrational excitation corresponding to the dip frequency. The dip intensities were estimated by fitting the data using the single Gaussian function. To estimate the relative conformer populations in MVK, vibrations 1 and 19 at 3103 and 2964 cm^−1^ for the s-*trans* and the s-*cis* conformers, respectively, were chosen. Then, the dip intensities were divided by FC-factors of 1.56 × 10^6^ (s-*trans*) and 1.88 × 10^6^ (s-*cis*) and IR absorption probabilities of 7.2 (s-*trans*) and 8.0 (s-*cis*). Thus, the relative populations of two conformers were determined as 90.6% (s-*trans*) and 9.4% (s-*cis*), which agree closely with previous values of 88% and 12%, which were determined by one-photon VUV-MATI spectroscopy.^9^ Similarly, we determined the relative conformer populations in CA by using vibrations 5 and 5 at 2724 (s-*trans*) and 2735 cm^−1^ (s-*cis*), assuming no Fermi resonance-induced changes in intensity. The corresponding FC factors and IR absorption probabilities were 0.38 and 635 (s-*trans*) and 0.37 and 1052 (s-*cis*), leading to the relative amounts of the two conformers in CA being calculated as 95.8% (s-*trans*) and 4.2% (s-*cis*), which are in excellent agreement with those reported for one-photon VUV-MATI spectroscopy: 96.5% and 3.5%, respectively.^10^ These results are summarized in Table 3.

**Table 3:** Comparison of the relative populations and stabilities of individual conformers in the MVK and CA isomers obtained in this work with those reported in previous studies

Method	s-*trans*	s-*cis*	Stability^d^
MVK			
This work	90.6%	9.4%	53 cm^−1^
VUV-MATI^a^	88%	12%	48 ± 18 cm^−1^
IR^b^	76%	24%	45 cm^−1^
IR and Raman^c^	69%	31%	162 cm^−1^

CA			
This work	95.8%	4.2%	706 cm^−1^
VUV-MATI^a^	96.5%	3.5%	634 cm^−1^
IR and Raman^c^	97%	3%	

a Values reported in ref. 9 and 10 for MVK and CA, respectively.

b Values reported in ref. 32.

c Values reported in ref. 33.

d Conformational stabilities between individual conformers determined from the characteristic peaks in the IR dip VUV-MATI spectra of MVK and CA at temperatures of 27 K and 298 K, respectively, utilizing Δ*H* = −*RT* ln *K* + *T*Δ*S*, where *K* was estimated by the relative populations and Δ*S* was determined by quantum chemical calculations.

The difference in the relative conformational preferences for the MVK and CA isomers can be attributed to the extent to which the highest occupied molecular orbitals, which consist of nonbonding orbitals on the oxygen atom in the carbonyl group interacting with the σ orbitals in the molecular plane, are stabilized. This depends on the position of the methyl group. Stabilization between the σ orbitals and lone-pair p orbital of the oxygen atom occurs through hyperconjugation. Therefore, the stability of the s-*cis* conformer in MVK is expected to be higher than that in CA because of the interaction between the carbonyl and methyl groups, as shown in Fig. 3 and 4.

## Conclusions

Conformers have similar vibrational structures both in the S_0_ and D_0_ states due to the comparable force fields between their nuclei. This has led to the continuous development of vibrational spectroscopic techniques to rigorously identify individual conformers of the designated molecule but only in the S_0_ state. Our results demonstrate that the IR hole-burn VUV-MATI spectra of individual conformers in the D_0_ state allow the VUV-MATI spectrum of a molecule to be rigorously deciphered, while the IR dip VUV-MATI spectra correspond to identifiable IR absorption spectra of individual conformers in the S_0_ state. Therefore, we expect that these conformer-specific vibrational spectroscopies will become a highly effective spectroscopic technique to elucidate the conformational structures of a molecule in the S_0_ and D_0_ states.

## Conflicts of interest

There are no conflicts to declare.

## Supplementary Material

RA-011-D1RA07397D-s001
