# Supplementary material for: Identification of individual conformers in C4H6O isomers using conformer-specific vibrational spectroscopy
Source: RSC Adv. 2021 Nov 29;11(60):38240–6. doi: 10.1039/d1ra07397d (PMC9044234; doi:10.1039/d1ra07397d)
Supplement: RA-011-D1RA07397D-s001 [file RA-011-D1RA07397D-s001.pdf]

Electronic Supplementary Material (ESI) for RSC Advances.
This journal is © The Royal Society of Chemistry 2021

Supplementary Information

Identification of Individual Conformers in C_4H_6O Isomers Using Conformer-Specific Vibrational Spectroscopy

Sung Man Park and Chan Ho Kwon*

Department of Chemistry and Institute for Molecular Science and Fusion Technology, Kangwon National University, Chuncheon 24341, Republic of Korea

* E-mail address: chkwon@kangwon.ac.kr (C. H. Kwon)

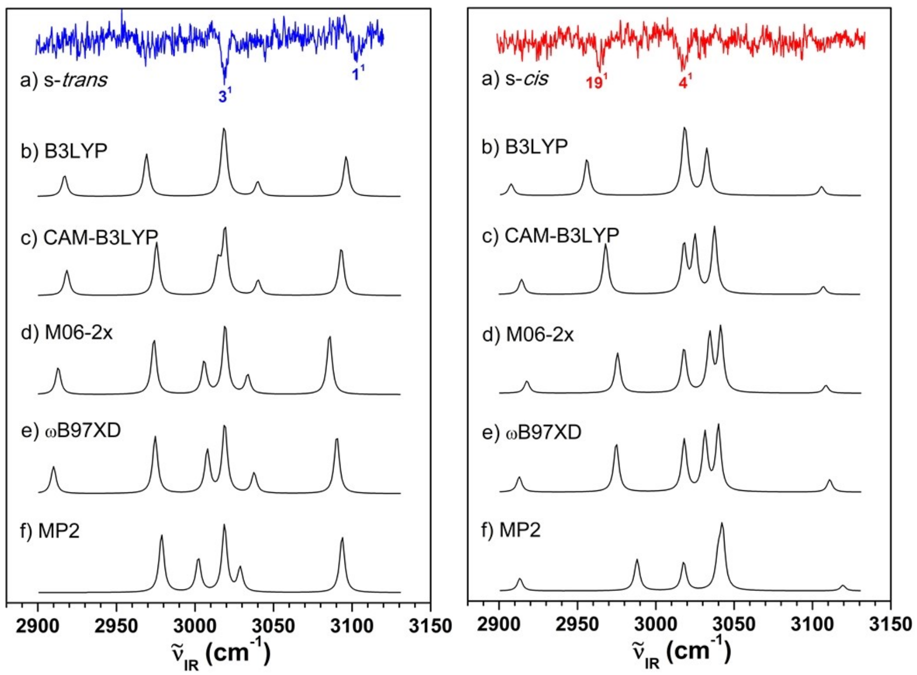

Figure S1. IR dip VUV-MATI spectra measured by monitoring the origin bands (77,861 and 78,216 cm^{-1}) of the s-*trans* (left) and the s-*cis* (right) conformers in MVK. IR spectra of the neutral s-*trans* (left) and neutral s-*cis* (right) conformers calculated at the (b) B3LYP, (c) CAM-B3LYP, (d) M06-2X, (e) ωB97XD, and (f) MP2 levels using the aug-cc-pVTZ basis set with s-*trans* (s-*cis*) scaling factors of 0.9606 (0.9612), 0.9525 (0.9546), 0.9478 (0.9525), 0.9513 (0.9554), and 0.9430 (0.9495), respectively.

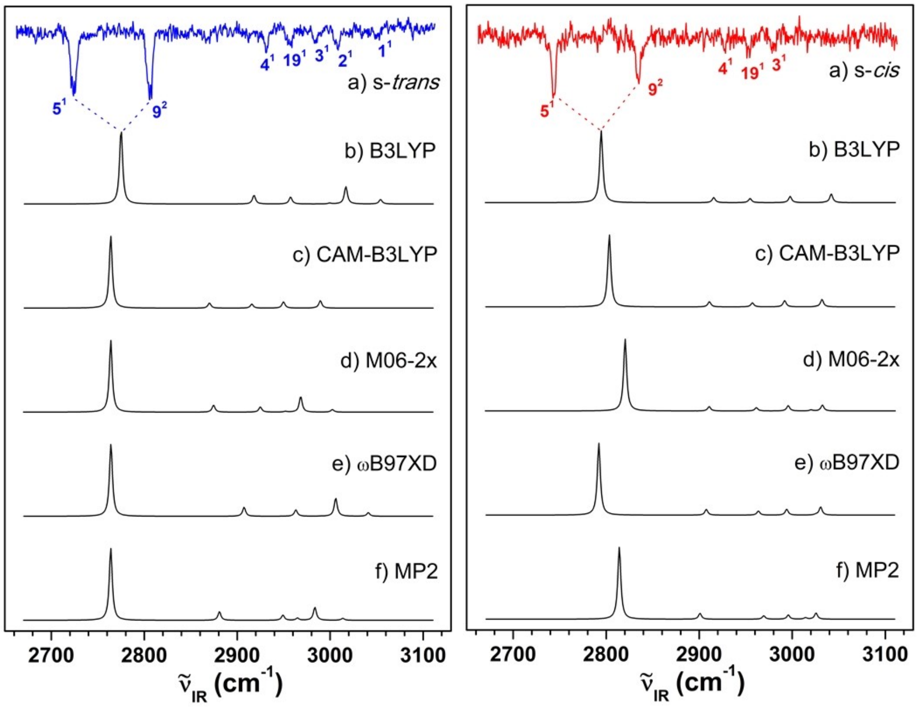

Figure S2. IR dip VUV-MATI spectra measured by monitoring the origin bands (78,638 and 78,734 cm^{-1}) of the s-*trans* (left) and the s-*cis* (right) conformers in CA. IR spectra of the neutral s-*trans* (left) and neutral s-*cis* (right) conformers calculated at the (b) B3LYP, (c) CAM-B3LYP, (d) M06-2X, (e) ωB97XD, and (f) MP2 levels using the aug-cc-pVTZ basis set, with s-*trans* (s-*cis*) scaling factors of 0.9667 (0.9667), 0.9421 (0.9555), 0.9395 (0.9519), 0.9544 (0.9549), and 0.9392 (0.9468), respectively.
